# Dynamic γδ T-cell Receptor remodeling and antigen-induced T-cell IFN-γ responses after SARS-CoV-2 infection during pregnancy: insights for γδ T-cell immunotherapy

**DOI:** 10.3389/fimmu.2026.1914308

**Published:** 2026-09-14

**Authors:** Yuhan Ke, Rongfeng Pan, Ling Li, Jing He, Yafang Li, Yuyuan Chen, Hongjie Liu, Jiawei Li, Muzi Luo, Rong Guo, Yan Chen, Danchun Chen, Chengfang Xu

**Affiliations:** 1 Department of Obstetrics and Gynecology, The Third Affiliated Hospital of Sun Yat-Sen University, Guangzhou, China; 2 Guangdong Second Provincial General Hospital, Guangzhou, China; 3 Department of Endocrinology, Guangdong Sanjiu Brain Hospital, Guangzhou, China; 4 Guangzhou Kingmed Center for Clinical Laboratory Co., Ltd., Guangzhou, China; 5 The Affiliated High School of South China Normal University, Guangzhou, China; 6 The First Affiliated Hospital of Gannan Medical University, Ganzhou, China; 7 Institute of Translational Medicine Zhuhai People’s Hospital, Zhuhai, China; 8 Pediatric and Adolescent Growth and Weight Management Center, The Third Affiliated Hospital of Sun Yat-Sen University, Guangzhou, China; 9 Children’s Medical Center, The Third Affiliated Hospital of Sun Yat-Sen University, Guangzhou, China

**Keywords:** IFN-γ, maternal-fetal immunity, PD-1, pregnancy, receptor remodeling, SARS-CoV-2, T-cell immunotherapy, γδ-T cells

## Abstract

**Introduction:**

Pregnancy requires coordinated immune adaptation to maintain fetal tolerance while preserving antiviral defense. However, the temporal dynamics of maternal cellular immunity and maternal-fetal antibody transfer after SARS-CoV-2 infection during pregnancy remain incompletely defined.

**Methods:**

We enrolled 52 pregnant women with laboratory-confirmed SARS-CoV-2 infection and stratified them by infection-to-delivery interval: <90 days, 90–120 days, and >120 days. Maternal peripheral blood mononuclear cells were analyzed by multiparametric flow cytometry. Basal and Omicron BA.5.2 antigen-induced IFN-γ production by CD3^+^, CD4^+^, and CD8^+^ T cells were assessed, and SARS-CoV-2-specific S1/RBD IgM and IgG levels were measured in paired maternal and cord blood by ELISA.

**Results:**

Baseline characteristics and major perinatal outcomes were broadly comparable among groups. Maternal T cells retained antigen-inducible IFN-γ responses, with the strongest responses observed in the 90–120-day group. Immune phenotyping revealed interval-associated remodeling of adaptive T-cell subsets, γδ T-cell receptor phenotypes, NK-cell subsets, and selected B-cell compartments. Cord blood IgM remained consistently low, providing no immunological evidence of intrauterine SARS-CoV-2 infection. In contrast, maternal and cord blood IgG levels were positively correlated, indicating preserved transplacental IgG transfer without clear time-dependent changes in transfer efficiency.

**Conclusions:**

These results extend current understanding of post-infection immune adaptation during pregnancy and provide an immunological basis for future longitudinal studies aimed at defining the timing and durability of maternal and neonatal immune protection.

## Introduction

Pregnancy is a complex physiological state in which the maternal immune system must maintain tolerance toward the semi-allogeneic fetus while preserving effective defense against exogenous pathogens (1). This process depends on finely regulated immune homeostasis; when this balance is disrupted, the risk of infection-related complications and adverse pregnancy outcomes may increase (2). Therefore, systematic evaluation of immune status and its dynamic changes during pregnancy is important for understanding maternal-fetal immune regulation and for optimizing clinical management.

Immune regulation during pregnancy involves broad remodeling of both innate and adaptive immunity, and functional changes in T lymphocytes are considered central to the maintenance of immune balance (3). The maternal-fetal interface is a key site of immune regulation (4, 5), with a microenvironment that changes markedly across gestational stages (6). In parallel, maternal IgG is selectively transported across the placenta to provide passive immune protection to the fetus (7), whereas IgM and IgA generally do not cross the placental barrier (8). Together, these mechanisms constitute the unique immune regulatory network of pregnancy.

Severe acute respiratory syndrome coronavirus 2 (SARS-CoV-2) infection has broad effects on the immune system. Infection can induce pronounced cellular and humoral immune responses (9), including activation of CD4^+^ and CD8^+^ T cells, production of effector cytokines such as IFN-γ, and generation of virus-specific antibodies (10, 11). Previous studies have shown that these immune responses are strongly time dependent (12), with their magnitude and functional status changing dynamically after infection (13, 14). In pregnancy, however, this process may be jointly influenced by immune tolerance mechanisms and endocrine regulation (15), and its temporal pattern remains insufficiently defined.

Current studies of immune responses after SARS-CoV-2 infection during pregnancy have mainly focused on humoral immunity and maternal-fetal antibody transfer (16). Although maternal virus-specific IgG can be transferred across the placenta, its transfer efficiency and neutralizing activity vary and are affected by the timing of infection and maternal immune status (17, 18). By contrast, cellular immunity, particularly T-cell functional status and its temporal changes, has not been systematically characterized (19). In addition, most studies have grouped participants according to infection status alone, with limited attention to the interval between infection and delivery as a key variable (20, 21). This limits a deeper understanding of the temporal features of the maternal immune response.

T lymphocytes play a central role in antiviral immunity by mediating antigen recognition, effector molecule production, and immune memory formation during viral clearance (22). After infection, a subset of T cells differentiates into memory cells capable of rapid responses upon re-exposure (23). Whether pregnancy-associated immune regulation influences the establishment and maintenance of this response remains unclear. Evidence suggests that the immune system during pregnancy may be functionally remodeled while retaining defensive capacity (24), but the specific manifestations and temporal pattern of this remodeling have not been fully elucidated. Accordingly, systematic investigation of T-lymphocyte function and its temporal dynamics after SARS-CoV-2 infection during pregnancy is important for understanding immune remodeling and assessing maternal-fetal risk. When combined with humoral immune features, particularly IgM and IgG levels and their maternal-fetal transfer, such analysis may provide a more comprehensive view of post-infection immune status during pregnancy.

The distinctive feature of the present study is that it evaluates SARS-CoV-2 infection during pregnancy from a temporal perspective rather than simply comparing infected and non-infected individuals. We included pregnant women with different infection-to-delivery intervals and analyzed maternal immune response profiles in a paired maternal and cord blood framework. By assessing T-cell functional status, immune cell phenotypes, antibody levels, and maternal-fetal antibody correspondence, this study aimed to delineate the time-dependent immune response after SARS-CoV-2 infection during pregnancy and to explore its relationship with maternal-fetal immune protection. Through the integration of cellular and humoral immune indicators, we further propose a dynamic immune framework for viral infection during pregnancy, providing a basis for individualized risk assessment and maternal-neonatal protection strategies.

## Materials and methods

### Study design and participants

Pregnant women with SARS-CoV-2 infection who attended the Third Affiliated Hospital of Sun Yat-sen University in Guangzhou, China, between December 2022 and May 2023 were enrolled. These women were confirmed to be pregnant after SARS-CoV-2 infection. The inclusion criteria were as follows: (1) age >=18 years; (2) singleton pregnancy; and (3) laboratory-confirmed SARS-CoV-2 infection, based on a positive nucleic acid test or antigen test. The exclusion criteria were as follows: (1) concurrent acute infectious diseases; (2) a history of or current immune-related diseases; (3) a history of severe underlying medical conditions; (4) long-term use of immunomodulatory or immunosuppressive medications before or during pregnancy; (5) severe pregnancy-related complications; and (6) SARS-CoV-2 infection occurring shortly after COVID-19 vaccination. A total of 52 infected pregnant women were included: 22 were infected less than 90 days before delivery, 16 were infected 90–120 days before delivery, and 14 were infected more than 120 days before delivery. The 90- and 120-day thresholds were specified *a priori* to create clinically interpretable approximate 3- and 4-month intervals, informed by the expected transition from early effector responses toward memory stabilization and by the need to retain analyzable subgroup sizes; they were not selected through outcome-based statistical optimization. All participants had received COVID-19 vaccination before the documented infection. Because spike/RBD-directed vaccine- and infection-derived responses could not be separated in this design, the findings are interpreted in the context of hybrid immunity. All procedures that involved human sample collection were approved by the ethics committee of the Third Affiliated Hospital of Sun Yat-sen University (Approval No.: II2023-089-01).

### Isolation of peripheral blood mononuclear cells and multiparametric flow cytometric analysis

Peripheral blood samples (5 mL) were collected from all participants before delivery into sodium heparin anticoagulant tubes. Peripheral blood mononuclear cells (PBMCs) were isolated by Ficoll density-gradient centrifugation and were immediately processed for surface phenotyping. Across the phenotyping panels, mouse anti-human fluorochrome-conjugated monoclonal antibodies included CD3, CD4, CD8, CD45, CD19, CD45RA, CCR7, CD28, CXCR5, PD-1, CXCR3, CCR4, CCR6, CD25, CD127, CD57, CD56, CD94, KIR, NKG2D, NKP30, NKP46, CD27, CD38, CD21, CD24, IgD, IgM, Vδ2, TCRγδ, and Vδ1. Samples were incubated with antibodies for 30 min at 4 °C, washed twice with PBS (Solarbio), and resuspended in 200 μL PBS. Data were acquired on a Cytek Aurora full-spectrum flow cytometer (Cytek Biosciences) and analyzed using FlowJo 10.1 software (Tree Star Inc., Ashland, OR, USA). Events were first restricted to the lymphocyte region on FSC-A versus SSC-A plots, followed by singlet discrimination using FSC-A versus FSC-H. A viability dye was not included in these surface-phenotyping panels; scatter- and singlet-based gating reduced debris and aggregates but was not considered equivalent to direct live/dead discrimination. CD3^+^CD56^+^ cells were reported as NKT-like cells rather than invariant NKT cells; NK cells were defined as CD3^-^CD56^+^ and subdivided by relative CD56 intensity; γδ T cells were defined as CD3^+^TCRγδ^+^ and further divided into Vδ1^+^ and Vδ2^+^ subsets; B cells were defined as CD45^+^CD19^+^; circulating Tfh-like cells were defined as CD4^+^CXCR5^+^PD-1^+^; and phenotypic Treg-like cells were defined as CD4^+^CD25^+^CD127low/^-^. Naïve, central-memory, effector-memory, and TEMRA T-cell phenotypes were classified using the established CD45RA/CCR7 combinations.

### Omicron BA.5.2 recombinant protein stimulation of PBMCs

Cryopreserved PBMC aliquots were thawed for the functional assay and seeded into 96-well plates at a density of 2×10^6^ cells/mL. Each sample was divided into an Omicron BA.5.2 recombinant protein-stimulated group and an unstimulated control group. Omicron BA.5.2 recombinant protein (2 μg/mL; Sino Biological) was added to the stimulation group, whereas an equal volume of RPMI 1640 medium (Gibco) was added to the control group. The cells were incubated at 37 °C. One hour after stimulation, GolgiStop (a monensin-containing protein-transport inhibitor; BD Biosciences) was added to all wells at a 1:1000 dilution and remained present during the subsequent 15h incubation at 37 °C.

### Assessment of PBMC surface molecules and cytokine secretion

After stimulation, PBMCs were collected, washed with PBS, centrifuged at 1200 rpm for 10 min, and the supernatant was discarded. Cells were resuspended in PBS and stained with anti-human CD3-APC-Cy7 (BD Biosciences), anti-human CD4-BV750 (BioLegend), anti-human CD8-BV570 (BioLegend), and FVS-Alexa Fluor 700 (BioLegend) for 30 min in the dark. FVS-negative events were gated as viable cells before downstream CD3, CD4, and CD8 analysis. After washing with PBS, cells were resuspended in permeabilization buffer (BD Biosciences), incubated for 40 min at 4 °C in the dark, washed with fixation buffer (BD Biosciences), and resuspended in fixation buffer. Then, anti-human IFN-γ-PE-Cy7 (BD Biosciences) was added. Subsequently, the cells were incubated overnight at 4 °C in the dark. Cells were washed twice with fixation buffer and resuspended in 200 μL fixation buffer. Data were acquired on a Cytek Aurora full-spectrum flow cytometer (Cytek Biosciences) and analyzed using FlowJo 10.1 software (Tree Star Inc.).

### ELISA

RBD-IgM and S1-IgM levels in maternal peripheral blood and umbilical cord blood were measured using commercial ELISA kits (Anti-SARS-CoV-2 Antibody IgM ELISA Kit [Spike RBD] and Anti-SARS-CoV-2 Antibody IgM ELISA Kit [Spike S1]; ACRO Biosystems). RBD-IgG and S1-IgG levels in maternal peripheral blood and umbilical cord blood were measured using commercial ELISA kits (Anti-SARS-CoV-2 Antibody IgG ELISA Kit [Spike RBD] and Anti-SARS-CoV-2 Antibody IgG ELISA Kit [Spike S1]; ACRO Biosystems).

### Statistical analysis

Data are presented as mean ± SD or as median with interquartile range (25th and 75th percentiles), depending on the distribution. Student’s t test and ANOVA were used for normally distributed data, whereas Mann-Whitney U test and Kruskal-Wallis H test were used for non-normally distributed data. Pearson’s or Spearman’s correlation coefficients were used to assess correlations for normally or non-normally distributed data, respectively. All data were analyzed using SPSS software version 25.0 and GraphPad Prism version 9.0. P values <0.05 were considered statistically significant.

## Results

### Baseline characteristics and perinatal outcomes across infection-to-delivery interval groups

A total of 52 pregnant women with laboratory-confirmed SARS-CoV-2 infection between December 2022 and May 2023 who met the inclusion criteria were enrolled. To ensure comparability among groups and to objectively evaluate the potential influence of the infection-to-delivery interval on perinatal outcomes, we systematically compared baseline clinical characteristics and perinatal outcomes. According to the interval from infection to delivery, participants were divided into three groups: <90 days, 90–120 days, and >120 days.

The three groups showed broadly comparable distributions of major demographic and baseline clinical characteristics, including maternal age, gravidity, parity, height, pre-pregnancy BMI and gestational weeks at infection (**Table 1**). Maternal age was similar among the <90-day, 90-120-day, and >120-day groups, and no significant intergroup differences were observed in gravidity, parity, height, or pre-pregnancy BMI, suggesting that the basic maternal characteristics were generally balanced across groups. In contrast, there were significant differences in gestational age at SARS-CoV-2 infection among the <90-day, 90-120-day, and >120-day groups (29.86 ± 1.73, 22.53 ± 1.39, and 21.57 ± 1.16 weeks, respectively; P<0.001). The differences were expected because the groups were defined according to the interval between infection and delivery. Therefore, the data was presented as a descriptive feature of the study design rather than interpreted as an independent biological finding.

**Table 1:** Baseline clinical characteristics of pregnant women with SARS-CoV-2 infection according to infection-to-delivery interval.

Characteristics	<90d (*n* = 22)	90-120d (*n* = 16)	>120d (*n* = 14)	*x*^2^/*F*	*P*
Age	32.18 ± 4.26	30.69 ± 2.36	32.21 ± 4.64	0.681	0.511
Gravida	1.59(1.00~2.00)	1.85(1.00~2.50)	2.07(1.00~3.00)	0.486	0.784
Parity	0.50(0~1.00)	0.54(0~1.00)	0.50(0~1.00)	0.046	0.977
Height (cm)	160.14 ± 4.50	156.23(152.50~161.00)	158.93 ± 4.71	4.720	0.094
Pre-pregnancy BMI (kg/m^2^)	22.15 ± 2.94	20.70 ± 3.62	20.62(18.80~21.96)	4.447	0.108
Infection gestational weeks (w)	29.86 ± 1.73	22.53 ± 1.39	21.57 ± 1.16	1.886	<0.001

Baseline demographic and clinical characteristics were compared among pregnant women grouped according to the interval between SARS-CoV-2 infection and delivery: <90 days, 90–120 days, and >120 days. Data are presented as mean ± SD or median (interquartile range), as appropriate. The χ²/F value and corresponding P value indicate intergroup comparisons. P < 0.05 was considered statistically significant. BMI, body mass index; d, days.

We next compared pregnancy-related complications and fetal/neonatal outcomes (**Table 2**). The incidences of miscarriage, gestational diabetes mellitus, hypertensive disorders of pregnancy, preterm birth, placental abnormalities, premature rupture of membranes, and postpartum hemorrhage did not differ significantly among the three groups. Most neonatal outcomes, including birth weight, Apgar score, referral to pediatrics, and neonatal hyperbilirubinemia, were also comparable across groups. Although the rate of fetal growth restriction/small for gestational age showed a nominal intergroup difference, this finding was not accompanied by consistent differences in birth weight or other major neonatal outcomes and should therefore be interpreted cautiously given the limited sample size.

**Table 2:** Maternal biochemical, coagulation, renal, and liver function parameters according to infection-to-delivery interval after SARS-CoV-2 infection during pregnancy.

Characteristics	<90d (*n* = 22)	90-120d (*n* = 16)	>120d (*n* = 14)	*x*^2^/*F*	*P*
K (mmol/L)	3.86 ± 0.26	3.93 ± 0.27	4.00 ± 0.20	1.271	0.290
Ca (mmol/L)	2.25 ± 0.15	2.38(2.29~2.43)	2.24 ± 0.14	3.850	0.146
Urea (mmol/L)	3.50 ± 0.89	3.33 ± 0.65	3.19 ± 0.95	0.594	0.556
CREAT (umol/L)	48.43 ± 9.91	45.41(43.00~50.50)	41.63 ± 8.85	3.886	0.143
PTset (s)	12.26 ± 0.48	12.24(11.90~12.45)	12.23 ± 0.40	0.307	0.858
Fibgl (g/L)	4.72 ± 0.62	4.68 ± 0.52	4.80 ± 0.67	0.130	0.878
APTTsec (s)	33.23 ± 2.32	31.81 ± 1.68	32.86 ± 2.74	0.631	0.536
TTsec (s)	17.86(16.18~17.73)	17.00 ± 1.37	16.99(15.88~18.50)	0.256	0.880
AST (U/L)	21.09(14.75~23.25)	18.31 ± 5.04	16.14(13.75~18.25)	3.831	0.147
ALT (U/L)	12.32(8.75~17.00)	13.00(9.50~15.50)	9.71 ± 3.36	2.113	0.348
ALB (g/L)	33.81 ± 2.07	33.24 ± 2.40	30.92 ± 1.85	8.396	0.001

Maternal laboratory indicators, including electrolytes, renal function, coagulation parameters, and liver function, were compared among pregnant women grouped according to the interval between SARS-CoV-2 infection and delivery: <90 days, 90–120 days, and >120 days. Data are presented as mean ± SD or median (interquartile range), as appropriate. The χ²/F value and corresponding P value indicate intergroup comparisons. P < 0.05 was considered statistically significant. K, potassium; Ca, calcium; CREAT, creatinine; PT, prothrombin time; FIB, fibrinogen; APTT, activated partial thromboplastin time; TT, thrombin time; AST, aspartate aminotransferase; ALT, alanine aminotransferase; ALB, albumin; d, days.

Further analyses of routine maternal laboratory indices, including liver and renal function, coagulation parameters, biochemical indicators, and complete blood counts, showed broadly comparable profiles across the three groups (**Supplementary Tables 1**, **2**). Similarly, neonatal cord blood gas parameters and metabolic screening results were generally comparable among groups (**Supplementary Table 3**). Although several individual indicators, including maternal albumin, cord blood PO_2_, and neonatal G6PD, showed statistically significant intergroup differences, these were isolated findings and were not accompanied by consistent changes in related clinical or laboratory parameters. These differences should therefore be interpreted cautiously and do not support a systematic pattern of maternal or neonatal laboratory abnormalities.

Taken together, with the exception of the expected difference in gestational age at infection resulting from the grouping strategy, the three groups were broadly comparable in their major maternal baseline characteristics, perinatal outcomes, and routine clinical laboratory indicators. No consistent interval-associated pattern was observed in major pregnancy complications or fetal and neonatal outcomes. This overall clinical comparability suggests that the observed immune differences were unlikely to be explained solely by major clinical imbalances among the groups.

### Interval-associated remodeling of maternal adaptive T-cell phenotypes after SARS-CoV-2 infection during pregnancy

To further clarify the effect of the infection-to-delivery interval on maternal adaptive immune status, we analyzed peripheral blood T-cell subsets by multiparametric flow cytometry (**Figure 1**). The overall proportions of CD3^+^ T cells among lymphocytes and CD4^+^ T cells among CD3^+^ T cells were comparable among the <90-day, 90–120-day, and >120-day groups, with no statistically significant differences (**Figures 1A, B**). These findings suggest that the infection-to-delivery interval did not substantially affect the overall abundance of circulating T cells or the major CD4^+^ T-cell compartment. The gating strategies used for phenotypic analysis of maternal immune cell subsets, including adaptive T-cell subsets, γδ T cells, NK cells, and B-cell compartments, are shown in **Supplementary Figures 1**, **2**.

**Figure 1:**
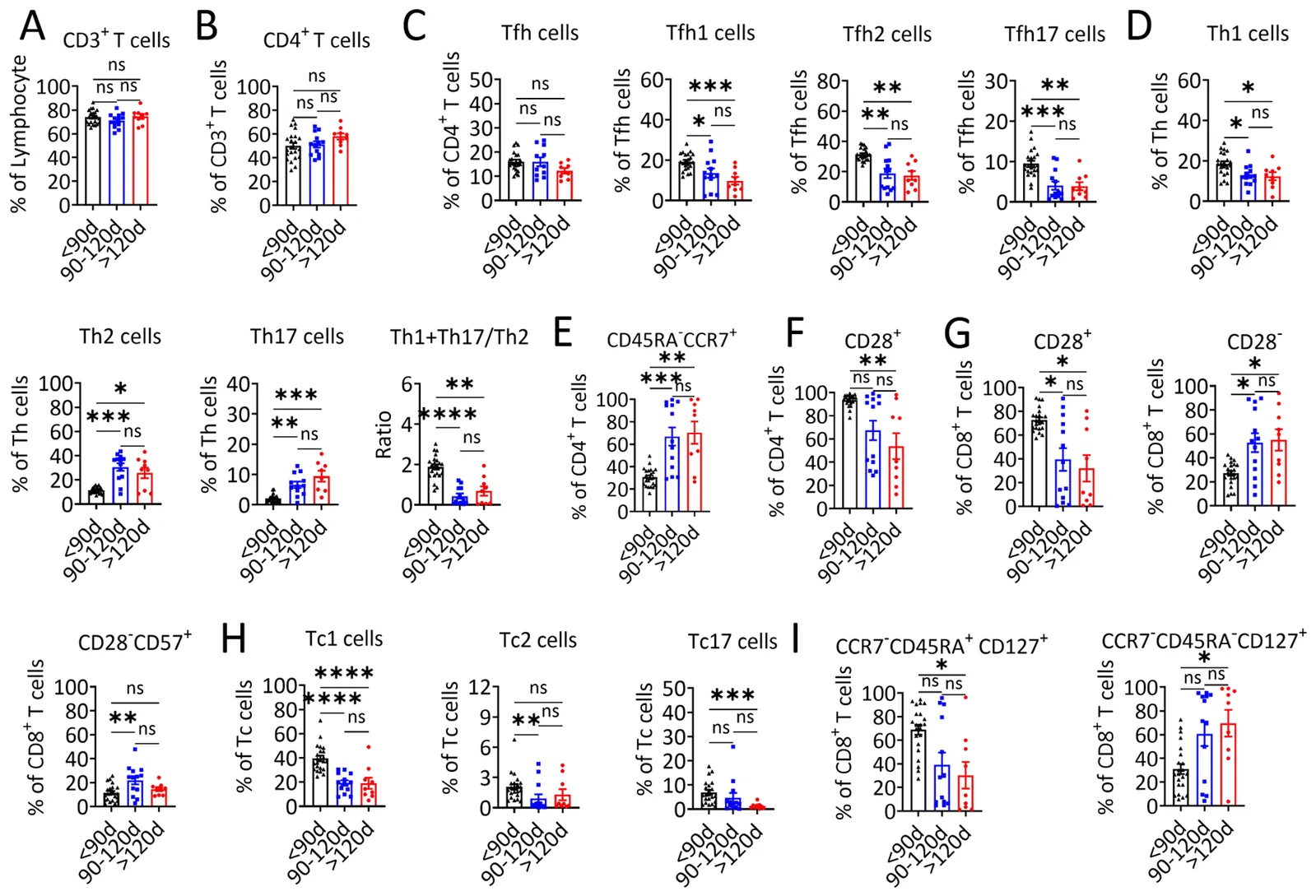
Interval-associated remodeling of maternal adaptive T-cell phenotypes after SARS-CoV-2 infection during pregnancy. Maternal peripheral blood T-cell phenotypes were analyzed by multiparametric flow cytometry in pregnant women grouped according to the infection-to-delivery interval: <90 days, 90–120 days, and >120 days. **(A)** Proportion of CD3^+^ T cells among lymphocytes. **(B)** Proportion of CD4^+^ T cells among CD3^+^ T cells. **(C)** Frequencies of circulating Tfh-like cells (CD4^+^CXCR5^+^PD-1^+^) and phenotypic Tfh1-, Tfh2-, and Tfh17-like subsets. **(D)** Frequencies of Th1, Th2, and Th17 cells, and the (Th1+Th17)/Th2 ratio. **(E)** Proportion of CD45RA^-^CCR7^+^ central memory CD4^+^ T cells. **(F)** Proportion of CD28^+^ CD4^+^ T cells. **(G)** Frequencies of CD28^+^, CD28^-^, and CD28^-^CD57^+^ CD8^+^ T-cell subsets. **(H)** Frequencies of Tc1, Tc2, and Tc17 cells. **(I)** Frequencies of CCR7^-^CD45RA^+^CD127^+^ and CCR7^-^CD45RA^-^CD127^+^ CD8^+^ T-cell subsets. Each dot represents one participant, and data are presented as mean ± SEM. *P < 0.05, **P < 0.01, ***P < 0.001, and ****P < 0.0001; ns, not significant.

We next examined Tfh-cell subsets and CD4^+^ T-cell polarization (**Figures 1C, D**). Although the total proportion of Tfh cells among CD4^+^ T cells did not differ significantly among the three groups, marked differences were observed in Tfh subset distribution. The proportions of Tfh1, Tfh2, and Tfh17 cells were significantly higher in the <90-day group than in the 90–120-day and/or >120-day groups, whereas no significant difference was observed between the 90–120-day and >120-day groups. Consistently, the (Th1+Th17)/Th2 ratio was significantly higher in the <90-day group than in the other two groups. Analysis of Th-cell polarization further showed that Th1 cells were more frequent in the <90-day group than in the 90–120-day and >120-day groups. In contrast, Th2 and Th17 cells were relatively lower in the <90-day group and increased in the later interval groups. These results suggest that early after infection, maternal CD4^+^ T cells displayed a more prominent Th1/Tfh-related immune activation pattern, followed by a relative shift toward Th2- and Th17-associated phenotypes at later infection-to-delivery intervals.

We further analyzed CD4^+^ T-cell memory and functional phenotypes (**Figures 1E, F**). The proportion of CD45RA^-^CCR7^+^ central memory CD4^+^ T cells was significantly lower in the <90-day group than in the 90–120-day and >120-day groups, suggesting an expansion of the central memory CD4^+^ T-cell compartment at later infection-to-delivery intervals. In contrast, CD28^+^CD4^+^ T cells were relatively more abundant in the <90-day group, with a significant reduction observed in the >120-day group. These findings indicate that CD4^+^ T cells may undergo a transition from an early activated or functional phenotype toward a more memory-associated phenotype over time after SARS-CoV-2 infection during pregnancy.

Based on the CD4^+^ T-cell subset analysis, we further assessed CD8^+^ T-cell functional, senescent, and effector differentiation phenotypes (**Figures 1G–I**). CD28^+^CD8^+^ T cells were more frequent in the <90-day group than in the 90–120-day and >120-day groups, whereas CD28^-^CD8^+^ T cells showed the opposite pattern, with higher levels in the 90–120-day and >120-day groups. In addition, CD28^-^CD57^+^ terminally differentiated or senescence-associated CD8^+^ T cells were significantly increased in the 90–120-day group compared with the <90-day group. These results indicate that CD8^+^ T cells gradually acquire more differentiated or senescence-associated phenotypes as the infection-to-delivery interval increases.

Among CD8^+^ effector subsets, Tc1 cells were significantly more frequent in the <90-day group than in both the 90–120-day and >120-day groups. Tc2 cells were also higher in the <90-day group than in the 90–120-day group, whereas Tc17 cells were higher in the <90-day group than in the >120-day group. Further analysis of memory-related CD8^+^ T-cell subsets showed that CCR7^-^CD45RA^+^CD127^+^ CD8^+^ T cells were more abundant in the <90-day group than in the >120-day group, whereas CCR7^-^CD45RA^-^CD127^+^ CD8^+^ T cells were less frequent in the <90-day group than in the >120-day group. These findings suggest that CD8^+^ T-cell effector and memory-related phenotypes vary dynamically with the infection-to-delivery interval.

Taken together, maternal adaptive T-cell phenotypes were not characterized by major changes in total T-cell abundance, but rather by interval-associated remodeling of CD4^+^ and CD8^+^ T-cell subset composition. The <90-day group showed more prominent Th1/Tfh-related and Tc1/Tc17-related effector features, whereas the 90–120-day and >120-day groups displayed increased memory-associated, differentiated, and senescence-related T-cell phenotypes. These results suggest a temporal transition from early effector immune activation toward later memory remodeling and functional adaptation after SARS-CoV-2 infection during pregnancy.

### Interval-associated remodeling of maternal γδ T-cell receptor phenotypes, NK-cell subsets, and B-cell compartments

In addition to αβ T cells, we analyzed NK cells and γδ T-cell subsets across the three infection-to-delivery interval groups (**Figures 2A–D**). For NK-cell-related populations, the proportions of CD3^+^CD56^+^ NKT-like cells and CD3^-^CD56^+^ NK cells among lymphocytes were comparable among groups (**Figure 2A**). However, the composition of NK-cell subsets showed modest interval-associated differences. The proportion of CD56high NK cells was significantly higher in the <90-day group than in the 90-120-day group, whereas CD56low NK cells showed a reciprocal increase in the 90-120-day group. No significant differences were observed in the CD56high/CD56low ratio or in CD94^+^KIR^-^ NK cells among the three groups. These findings suggest that the infection-to-delivery interval did not markedly affect the overall NK-cell frequency, but may be associated with a mild shift in CD56-defined NK-cell subset composition.

**Figure 2:**
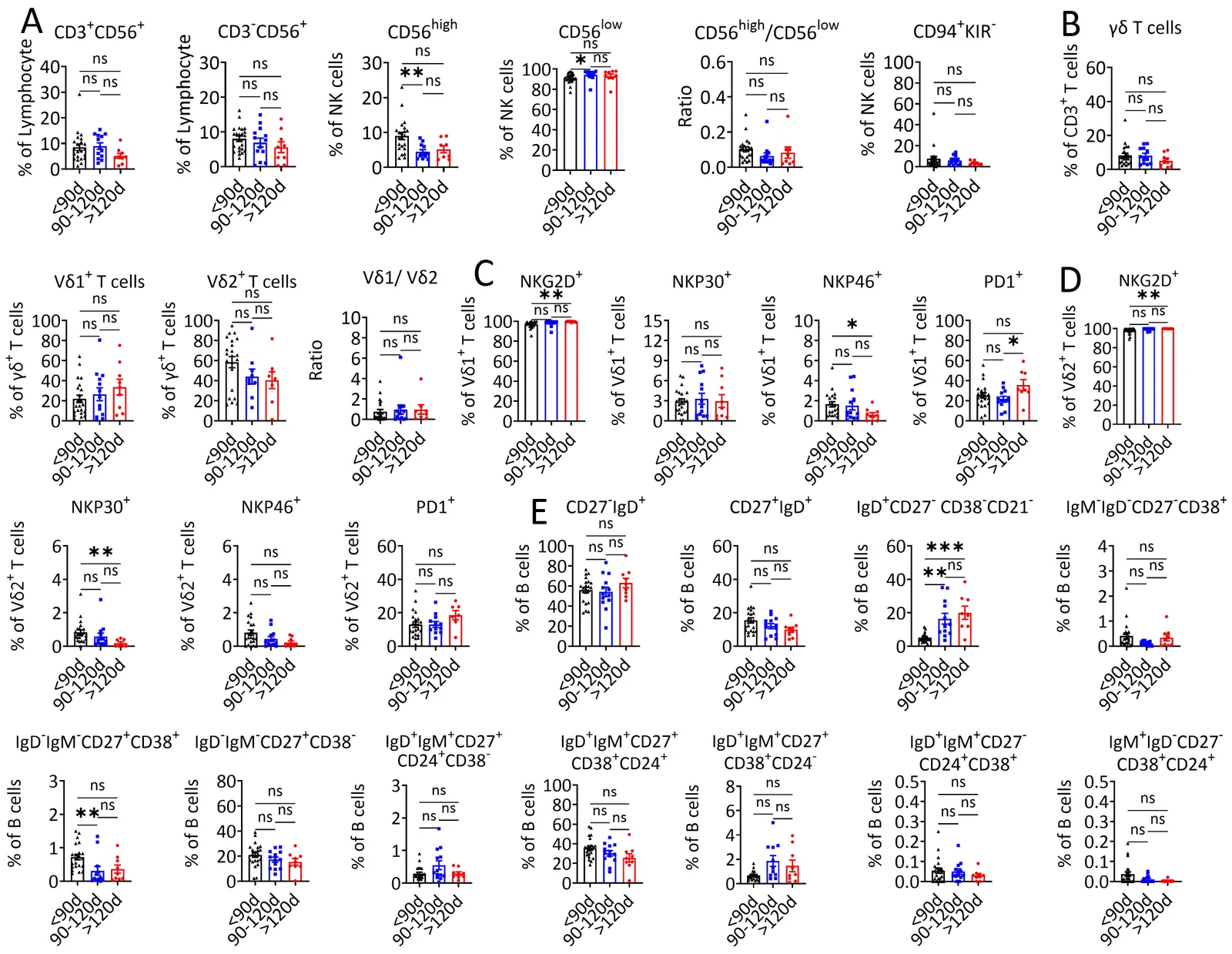
Interval-associated remodeling of maternal NK cells, γδ T cells, and B-cell subsets after SARS-CoV-2 infection during pregnancy. Maternal peripheral blood NK-cell subsets, γδ T-cell subsets, γδ T-cell receptor phenotypes, and B-cell compartments were analyzed by multiparametric flow cytometry in pregnant women grouped according to the infection-to-delivery interval: <90 days, 90–120 days, and >120 days. **(A)** Frequencies of CD3^+^CD56^+^ NKT-like cells and CD3^-^CD56^+^ NK cells among lymphocytes, and the proportions of CD56^high, CD56^low, CD56^high/CD56^low ratio, and CD94^+^KIR^-^ NK cells. **(B)** Frequencies of total γδ T cells among CD3^+^ T cells, Vδ1^+^ and Vδ2^+^ T-cell subsets among γδ T cells, and the Vδ1/Vδ2 ratio. **(C)** Expression of NKG2D, NKP30, NKP46, and PD-1 on Vδ1^+^ T cells. **(D)** Expression of NKG2D, NKP30, NKP46, and PD-1 on Vδ2^+^ T cells. **(E)** Frequencies of B-cell subsets defined by IgD, IgM, CD27, CD38, CD21, and CD24 expression among total B cells. Each dot represents one participant, and data are presented as mean ± SEM. *P < 0.05, **P < 0.01, ***P < 0.001; ns, not significant.

We next focused on the γδ T-cell compartment. The overall frequency of γδ T cells among CD3^+^ T cells did not differ significantly among the <90-day, 90-120-day, and >120-day groups (**Figure 2B**). Similarly, the proportions of Vδ1^+^ T cells and Vδ2^+^ T cells within total γδ T cells, as well as the Vδ1/Vδ2 ratio, remained broadly comparable among groups. These results suggest that the infection-to-delivery interval did not substantially alter the overall abundance or major subset composition of circulating γδ T cells.

Although the global γδ T-cell distribution was relatively stable, receptor-level phenotypic analysis revealed interval-associated remodeling within both Vδ1^+^ and Vδ2^+^ T-cell subsets. Among Vδ1^+^ T cells, NKG2D was highly expressed across all groups, but its expression was significantly lower in the >120-day group than in the <90-day group (**Figure 2C**). NKP30 expression on Vδ1^+^ T cells did not differ significantly among groups, whereas NKP46^+^ Vδ1^+^ T cells were reduced in the >120-day group compared with the <90-day group. In contrast, PD-1 expression on Vδ1^+^ T cells was significantly higher in the >120-day group than in the 90-120-day group. These findings indicate that Vδ1^+^ T cells may undergo a gradual phenotypic shift over time after SARS-CoV-2 infection, characterized by decreased expression of activating receptors and relatively increased expression of the inhibitory receptor PD-1.

Similar phenotypic changes were also observed in Vδ2^+^ T cells (**Figure 2D**). NKG2D expression remained high in all three groups, but was significantly lower in the >120-day group than in the <90-day group. NKP30^+^ Vδ2^+^ T cells were also significantly reduced in the >120-day group compared with the <90-day group. In contrast, NKP46 and PD-1 expression on Vδ2^+^ T cells did not show significant intergroup differences. Together, these results suggest that the main changes in γδ T cells after SARS-CoV-2 infection during pregnancy are not reflected by changes in total γδ T-cell frequency or Vδ1/Vδ2 subset distribution, but rather by functional receptor remodeling, particularly involving NKG2D, NKP30, NKP46, and PD-1 expression.

To further assess the cellular basis of maternal humoral immunity after SARS-CoV-2 infection during pregnancy, we examined B-cell subsets in maternal peripheral blood across different infection-to-delivery interval groups (**Figure 2E**). The proportions of CD27^-^IgD^+^ B cells and CD27^+^IgD^+^ B cells were comparable among groups. In contrast, IgD^+^CD27^-^CD38^-^CD21^-^ B cells were significantly less frequent in the <90-day group than in the 90-120-day and >120-day groups, whereas no significant difference was observed between the latter two groups. The IgM^-^IgD^-^CD27^-^CD38^+^ B-cell subset did not differ significantly among groups. Among plasma-cell-related or activated B-cell subsets, IgD^-^IgM^-^CD27^+^CD38^+^ B cells were significantly more frequent in the <90-day group than in the 90-120-day group, while no significant difference was observed between the 90-120-day and >120-day groups. Other B-cell subsets, including IgD^-^IgM^-^CD27^+^CD38^-^, IgD^+^IgM^+^CD27^+^CD24^+^CD38^-^, IgD^+^IgM^+^CD27^+^CD38^+^CD24^+^, IgD^+^IgM^+^CD27^+^CD38^+^CD24^-^, IgD^+^IgM^+^CD27^-^CD24^+^CD38^+^, and IgM^+^IgD^-^CD27^-^CD38^+^CD24^+^ B cells, showed no significant inter-group differences.

Taken together, these findings indicate that maternal innate-like lymphocyte and B-cell compartments are dynamically remodeled after SARS-CoV-2 infection during pregnancy. In particular, γδ T cells showed stable overall abundance and preserved Vδ1/Vδ2 subset distribution, but displayed evident receptor-level changes, suggesting that functional remodeling rather than numerical redistribution may be a major feature of γδ T-cell responses across different infection-to-delivery intervals. Meanwhile, NK-cell changes were mainly reflected by a mild shift in CD56high and CD56low subsets, whereas B-cell alterations were limited to selected subsets, including IgD^+^CD27^-^CD38^-^CD21^-^ B cells and IgD^-^IgM^-^CD27^+^CD38^+^ B cells.

### Time-dependent maternal T-cell IFN-γ responses after SARS-CoV-2 infection during pregnancy

To evaluate maternal T-cell functional responses after SARS-CoV-2 infection during pregnancy, we analyzed IFN-γ production in major T-cell populations of maternal PBMCs with or without the stimulation by Omicron BA.5.2 recombinant protein. Participants were compared according to the interval between infection and delivery. **Supplementary Figure 3** shows the gating strategy used to identify IFN-γ-producing CD3^+^, CD4^+^, and CD8^+^ T cells under unstimulated and antigen-stimulated conditions.

Under unstimulated conditions, the frequencies of IFN-γ^+^ cells within the CD3^+^, CD4^+^, and CD8^+^ T-cell populations were generally low (**Figure 3A**). Nevertheless, modest differences were observed among the three infection-to-delivery interval groups (**Figure 3D**). The basal frequencies of IFN-γ^+^ CD3^+^ and CD4^+^ T cells were significantly higher in the 90–120-day group than in the >120-day group. No significant differences were observed between the <90-day and 90–120-day groups or between the <90-day and >120-day groups. The basal frequency of IFN-γ^+^ CD8^+^ T cells also tended to be higher in the 90–120-day group, although the differences among groups did not reach statistical significance.

**Figure 3:**
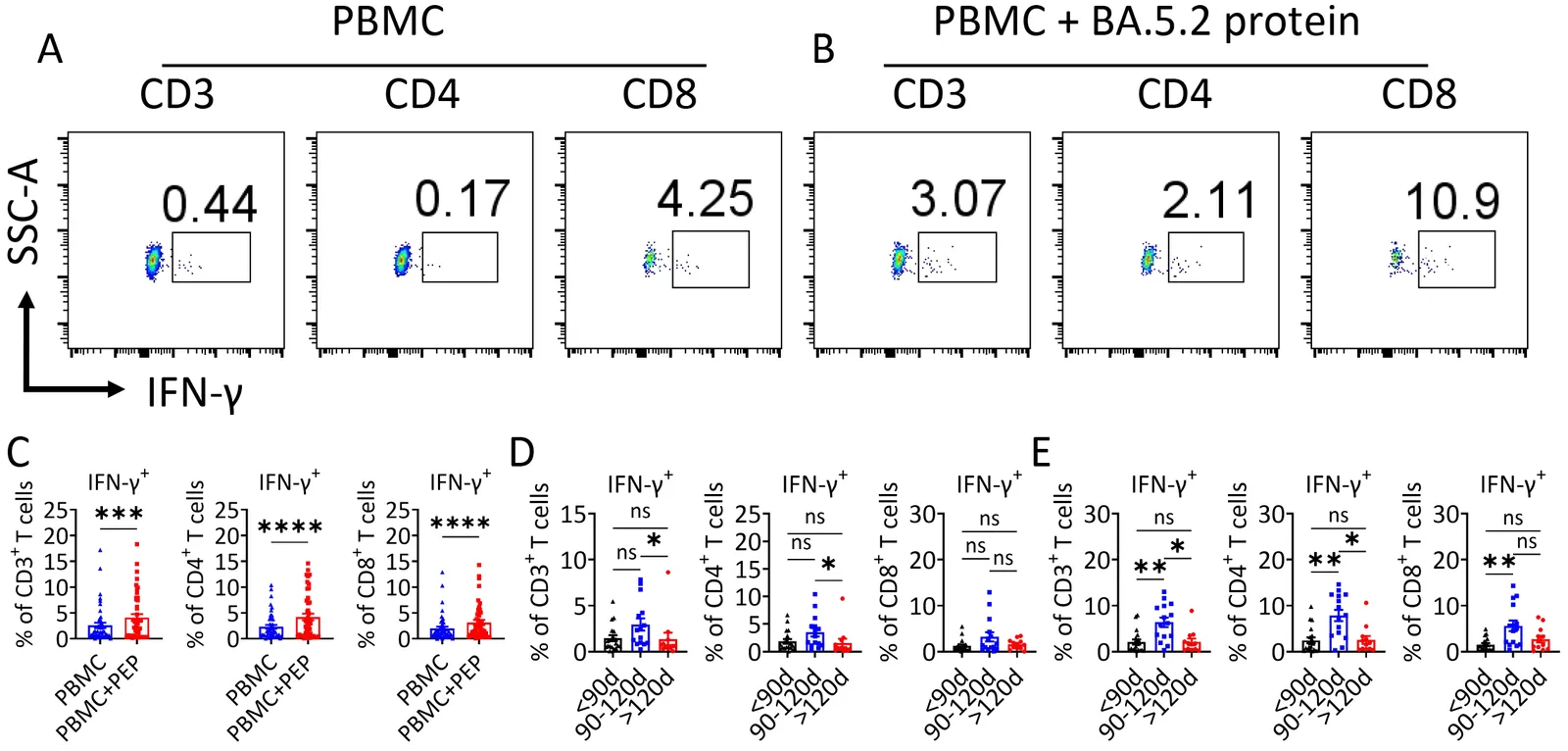
Maternal T-cell IFN-γ responses before and after SARS-CoV-2 Omicron BA.5.2 antigen stimulation. Maternal PBMCs were cultured under unstimulated conditions or stimulated with SARS-CoV-2 Omicron BA.5.2 recombinant protein antigen, and IFN-γ production by CD3^+^, CD4^+^, and CD8^+^ T cells was analyzed by flow cytometry. **(A)** Representative flow cytometry plots showing basal IFN-γ expression in CD3^+^, CD4^+^, and CD8^+^ T cells under unstimulated conditions. **(B)** Representative flow cytometry plots showing IFN-γ expression in CD3^+^, CD4^+^, and CD8^+^ T cells after Omicron BA.5.2 antigen stimulation. **(C)** Comparison of IFN-γ^+^ CD3^+^, CD4^+^, and CD8^+^ T-cell frequencies between unstimulated and antigen-stimulated conditions. **(D)** Comparison of basal IFN-γ^+^ CD3^+^, CD4^+^, and CD8^+^ T-cell frequencies among the <90-day, 90–120-day, and >120-day infection-to-delivery interval groups. **(E)** Comparison of antigen-induced IFN-γ^+^ CD3^+^, CD4^+^, and CD8^+^ T-cell frequencies among the three infection-to-delivery interval groups. Each dot represents one participant, and data are presented as mean ± SEM. *P < 0.05, **P < 0.01, ***P < 0.001, and ****P < 0.0001; ns, not significant.

We next assessed the capacity of maternal T cells to respond to viral antigen re-exposure by stimulating PBMCs *in vitro* with Omicron BA.5.2 recombinant protein. Representative flow-cytometry plots showed the increases in IFN-γ^+^ CD3^+^, CD4^+^, and CD8^+^ T-cell populations following antigen stimulation compared with the corresponding unstimulated samples (**Figures 3A, B**). Quantitative analysis further confirmed that stimulation significantly increased the frequencies of IFN-γ^+^ cells in all three T-cell populations (**Figure 3C**). These results indicate that maternal peripheral blood T cells retain antigen-inducible IFN-γ responsiveness after SARS-CoV-2 infection during pregnancy.

We further compared antigen-induced IFN-γ responses among the three infection-to-delivery interval groups (**Figure 3E**). The frequency of IFN-γ^+^ CD3^+^ T cells was significantly higher in the 90–120-day group than in both the <90-day and >120-day groups, whereas no significant difference was observed between the <90-day and >120-day groups. A similar result was observed for CD4^+^ T cells, with the highest response detected in the 90–120-day group. Among CD8^+^ T cells, the frequency of IFN-γ^+^ cells was significantly higher in the 90–120-day group than in the <90-day group, while the difference between the 90–120-day and >120-day groups was not statistically significant.

Taken together, maternal T cells retained IFN-γ responsiveness to Omicron BA.5.2 antigen after SARS-CoV-2 infection during pregnancy, with the strongest responses in the 90–120-day group, especially among CD3^+^ and CD4^+^ T cells. These findings reveal an interval-associated pattern of maternal cellular immunity and identify a potentially active window of antiviral T-cell responsiveness before delivery.

### Temporal profiles and maternal-fetal distribution of SARS-CoV-2-specific IgM and IgG responses

IgM is generally regarded as an important immunological marker of acute infection (25). Owing to its large molecular size, IgM usually does not efficiently cross the maternal-fetal barrier into the fetal circulation (26). In this study, S1-IgM and RBD-IgM optical density (OD) values were measured in maternal peripheral blood and matched cord blood from pregnant women with SARS-CoV-2 infection to evaluate the temporal features of maternal IgM responses and the distribution of IgM in cord blood.

We first compared SARS-CoV-2-specific IgM levels between maternal peripheral blood and paired cord blood. For S1-IgM, maternal peripheral blood showed significantly higher OD values than cord blood in the overall cohort and in each infection-to-delivery interval group (**Figure 4A**). A similar pattern was observed for RBD-IgM, with markedly higher OD values in maternal peripheral blood than in cord blood both in the overall population and within the <90-day, 90-120-day, and >120-day groups (**Figure 4C**). These results indicate that SARS-CoV-2-specific IgM responses were mainly detected in the maternal circulation, whereas cord blood IgM remained at very low levels across all groups.

**Figure 4:**
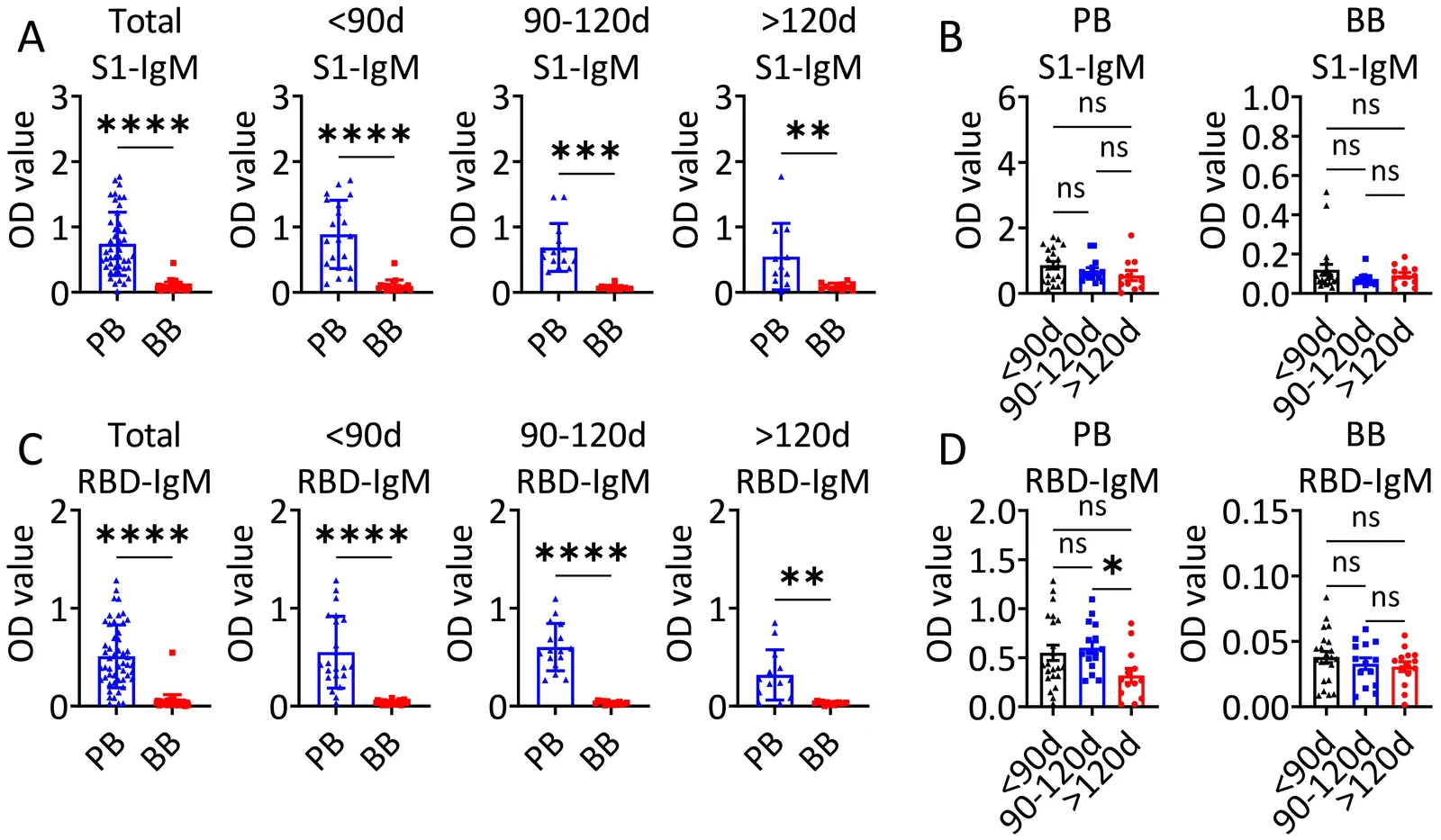
Maternal and cord blood SARS-CoV-2-specific IgM responses after infection during pregnancy. SARS-CoV-2-specific S1-IgM and RBD-IgM levels were measured in paired maternal peripheral blood and cord blood samples by ELISA and expressed as optical density **(OD)** values. Pregnant women were grouped according to the infection-to-delivery interval: <90 days, 90–120 days, and >120 days. **(A)** Comparison of S1-IgM OD values between maternal peripheral blood and cord blood in the overall cohort and within each infection-to-delivery interval group. **(B)** Intergroup comparison of S1-IgM OD values in maternal peripheral blood and cord blood. **(C)** Comparison of RBD-IgM OD values between maternal peripheral blood and cord blood in the overall cohort and within each infection-to-delivery interval group. **(D)** Intergroup comparison of RBD-IgM OD values in maternal peripheral blood and cord blood. PB, maternal peripheral blood; BB, cord blood. Each dot represents one participant, and data are presented as mean ± SEM. *P < 0.05, **P < 0.01, ***P < 0.001, and ****P < 0.0001; ns, not significant.

We next analyzed whether S1-IgM and RBD-IgM levels varied according to the infection-to-delivery interval. For S1-IgM, no significant intergroup differences were observed in either maternal peripheral blood or cord blood among the <90-day, 90-120-day, and >120-day groups (**Figure 4B**). The overall distribution of S1-IgM remained relatively stable without a clear time-dependent trend. For RBD-IgM, maternal peripheral blood OD values showed a time-related difference, with significantly lower levels in the >120-day group than in the 90-120-day group (**Figure 4D**). In contrast, cord blood RBD-IgM remained low and showed no significant differences among the three interval groups.

We further evaluated SARS-CoV-2-specific IgG responses in maternal peripheral blood and matched cord blood. For S1-IgG, cord blood OD values were significantly higher than maternal peripheral blood values in the overall cohort, while subgroup analysis showed a significant maternal-cord difference in the 90-120-day group but not in the <90-day or >120-day groups (**Figure 5A**). However, when S1-IgG levels were compared across different infection-to-delivery interval groups, no significant differences were observed in either maternal peripheral blood or cord blood (**Figure 5B**). The cord blood/maternal peripheral blood ratio for S1-IgG also showed no significant intergroup difference, suggesting that S1-IgG transfer was broadly maintained across different intervals.

**Figure 5:**
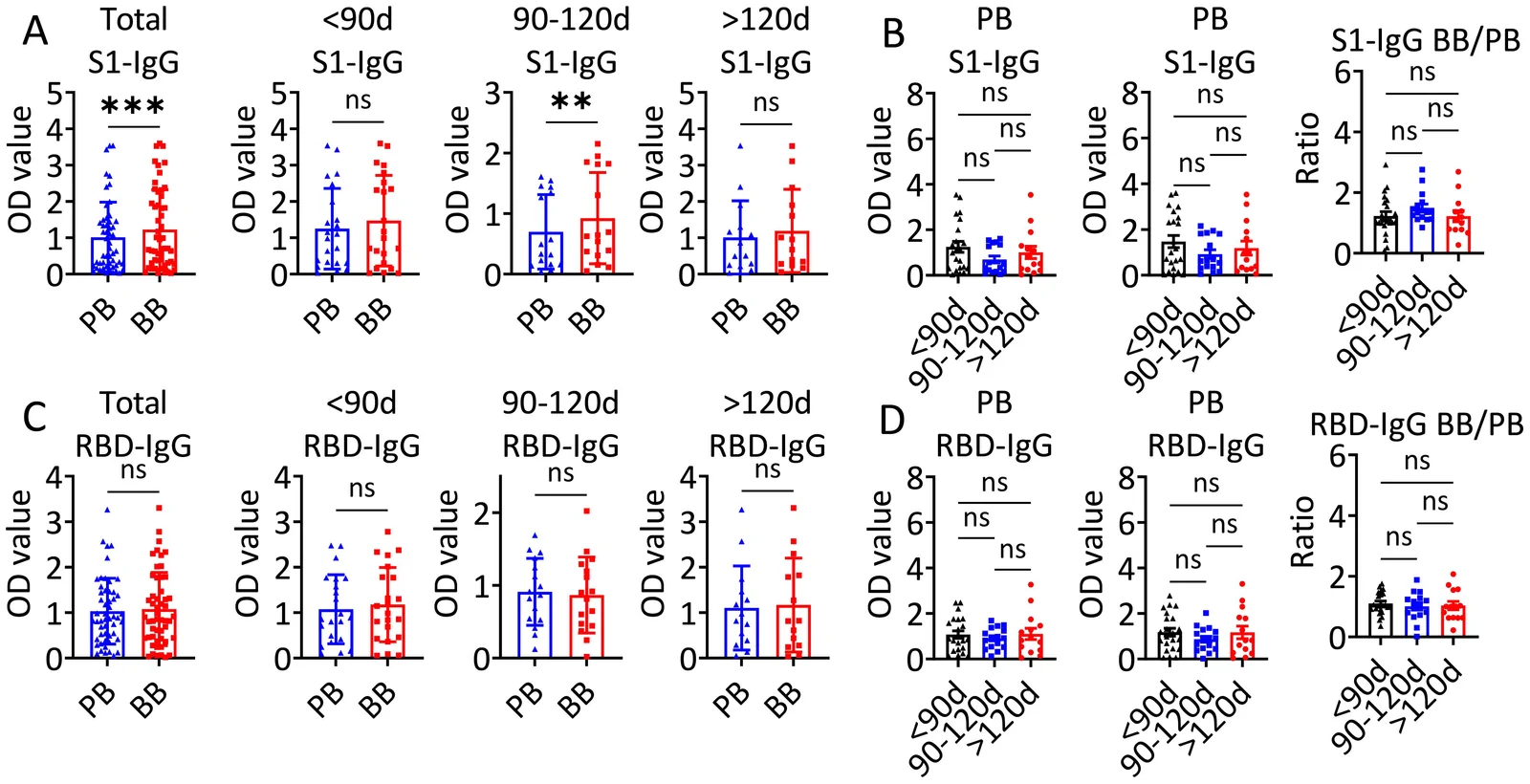
Maternal and cord blood SARS-CoV-2-specific IgG responses after infection during pregnancy. SARS-CoV-2-specific S1-IgG and RBD-IgG levels were measured in paired maternal peripheral blood and cord blood samples by ELISA and expressed as optical density **(OD)** values. Pregnant women were grouped according to the infection-to-delivery interval: <90 days, 90–120 days, and >120 days. **(A)** Comparison of S1-IgG OD values between maternal peripheral blood and cord blood in the overall cohort and within each infection-to-delivery interval group. **(B)** Intergroup comparison of S1-IgG OD values in maternal peripheral blood, cord blood, and the cord blood/maternal peripheral blood ratio. **(C)** Comparison of RBD-IgG OD values between maternal peripheral blood and cord blood in the overall cohort and within each infection-to-delivery interval group. **(D)** Intergroup comparison of RBD-IgG OD values in maternal peripheral blood, cord blood, and the cord blood/maternal peripheral blood ratio. PB, maternal peripheral blood; BB, cord blood. Each dot represents one participant, and data are presented as mean ± SEM. **P < 0.01 and ***P < 0.001; ns, not significant.

For RBD-IgG, no significant difference was observed between maternal peripheral blood and cord blood OD values, either in the overall cohort or within each infection-to-delivery interval group (**Figure 5C**). Similarly, maternal RBD-IgG levels, cord blood RBD-IgG levels, and the cord blood/maternal peripheral blood RBD-IgG ratio did not differ significantly among the three interval groups (**Figure 5D**). These findings indicate that RBD-IgG levels and maternal-fetal distribution were relatively stable across the different infection-to-delivery intervals.

Taken together, these results demonstrate distinct maternal-fetal distribution patterns of SARS-CoV-2-specific IgM and IgG after infection during pregnancy. SARS-CoV-2-specific IgM was predominantly detected in maternal peripheral blood, while cord blood IgM remained consistently low, providing no immunological evidence of fetal intrauterine SARS-CoV-2 infection in the included participants. In contrast, SARS-CoV-2-specific IgG was detectable in both maternal and cord blood, with broadly preserved maternal-fetal distribution and no clear time-dependent differences in transfer ratios. These findings suggest that maternal IgM responses, particularly RBD-IgM, may decline at later time intervals, whereas maternal-fetal IgG transfer remains relatively stable after SARS-CoV-2 infection during pregnancy.

### Maternal-cord blood distribution and temporal stability of SARS-CoV-2-specific IgM and IgG levels

Significant differences in SARS-CoV-2-specific IgM levels were observed between maternal peripheral blood and cord blood, both in the entire cohort and within each infection-to-delivery interval group. For both S1-IgM and RBD-IgM, maternal peripheral blood showed markedly higher OD values than matched cord blood, whereas cord blood IgM levels remained consistently low (**Figures 4A, C**). These findings suggest that SARS-CoV-2-specific IgM responses were mainly detected in the maternal circulation and that cord blood IgM did not reach levels clearly suggestive of fetal IgM positivity. Therefore, no immunological evidence supporting fetal intrauterine SARS-CoV-2 infection was observed in the included participants.

Because IgG can be transferred from mother to fetus through the placenta, its level reflects not only the persistence of maternal humoral immunity after infection during pregnancy but also the acquisition of passive immunity by the fetus. We therefore measured and compared S1-IgG and RBD-IgG OD values in maternal peripheral blood and matched cord blood across the different infection-to-delivery interval groups.

For S1-IgG, cord blood OD values were significantly higher than maternal peripheral blood values in the overall cohort (**Figure 5A**). Subgroup analysis showed that this maternal-cord blood difference was significant in the 90-120-day group, whereas no significant differences were observed in the <90-day or >120-day groups. However, when S1-IgG levels were compared among the three infection-to-delivery interval groups, no significant differences were observed in maternal peripheral blood, cord blood, or the cord blood/maternal peripheral blood ratio (**Figure 5B**). These results suggest that S1-IgG was efficiently detected in the fetal circulation, but its overall maternal and cord blood levels did not show a clear interval-dependent change.

For RBD-IgG, no significant difference was observed between maternal peripheral blood and cord blood OD values, either in the overall cohort or within each infection-to-delivery interval group (**Figure 5C**). Further intergroup comparisons showed that maternal RBD-IgG levels, cord blood RBD-IgG levels, and the cord blood/maternal peripheral blood ratio were all comparable among the <90-day, 90-120-day, and >120-day groups (**Figure 5D**). These findings indicate that RBD-IgG levels and maternal-fetal distribution remained relatively stable across different infection-to-delivery intervals.

Taken together, SARS-CoV-2-specific IgM and IgG showed distinct maternal-fetal distribution patterns after infection during pregnancy. IgM was predominantly detected in maternal peripheral blood and remained low in cord blood, supporting the limited transplacental transfer of IgM and providing no evidence of fetal intrauterine infection. In contrast, SARS-CoV-2-specific IgG was detectable in both maternal and cord blood, with S1-IgG showing higher levels in cord blood than in maternal blood in the overall cohort and in the 90-120-day group. However, neither S1-IgG nor RBD-IgG showed significant differences among infection-to-delivery interval groups in maternal blood, cord blood, or transfer ratio. These results suggest that maternal-fetal IgG transfer was broadly preserved after SARS-CoV-2 infection during pregnancy, without clear time-dependent changes across the examined intervals.

### Maternal-fetal transfer stability and correlation of SARS-CoV-2-specific IgG

To further evaluate the transfer characteristics and maternal-fetal correspondence of SARS-CoV-2-specific IgG, we analyzed the cord blood/maternal peripheral blood OD ratios for S1-IgG and RBD-IgG and performed correlation analyses between paired maternal peripheral blood and cord blood IgG levels.

As shown above, the cord blood/maternal peripheral blood OD ratios for both S1-IgG and RBD-IgG did not differ significantly among the <90-day, 90-120-day, and >120-day groups, and no clear increasing or decreasing trend was observed with the infection-to-delivery interval. These findings suggest that the overall maternal-fetal transfer efficiency of SARS-CoV-2-specific IgG remained relatively stable across different post-infection intervals.

We next examined the correlation between maternal peripheral blood and cord blood IgG levels. For S1-IgG (**Figure 6A**), a strong positive correlation was observed between maternal peripheral blood and cord blood in the overall cohort (P<0.0001, R²=0.8511). This correlation remained significant within each infection-to-delivery interval group, including the <90-day group (P<0.0001, R²=0.8508), the 90-120-day group (P<0.0001, R²=0.9236), and the >120-day group (P<0.0001, R²=0.799). These results indicate a close maternal-fetal correspondence of S1-IgG levels after SARS-CoV-2 infection during pregnancy.

**Figure 6:**
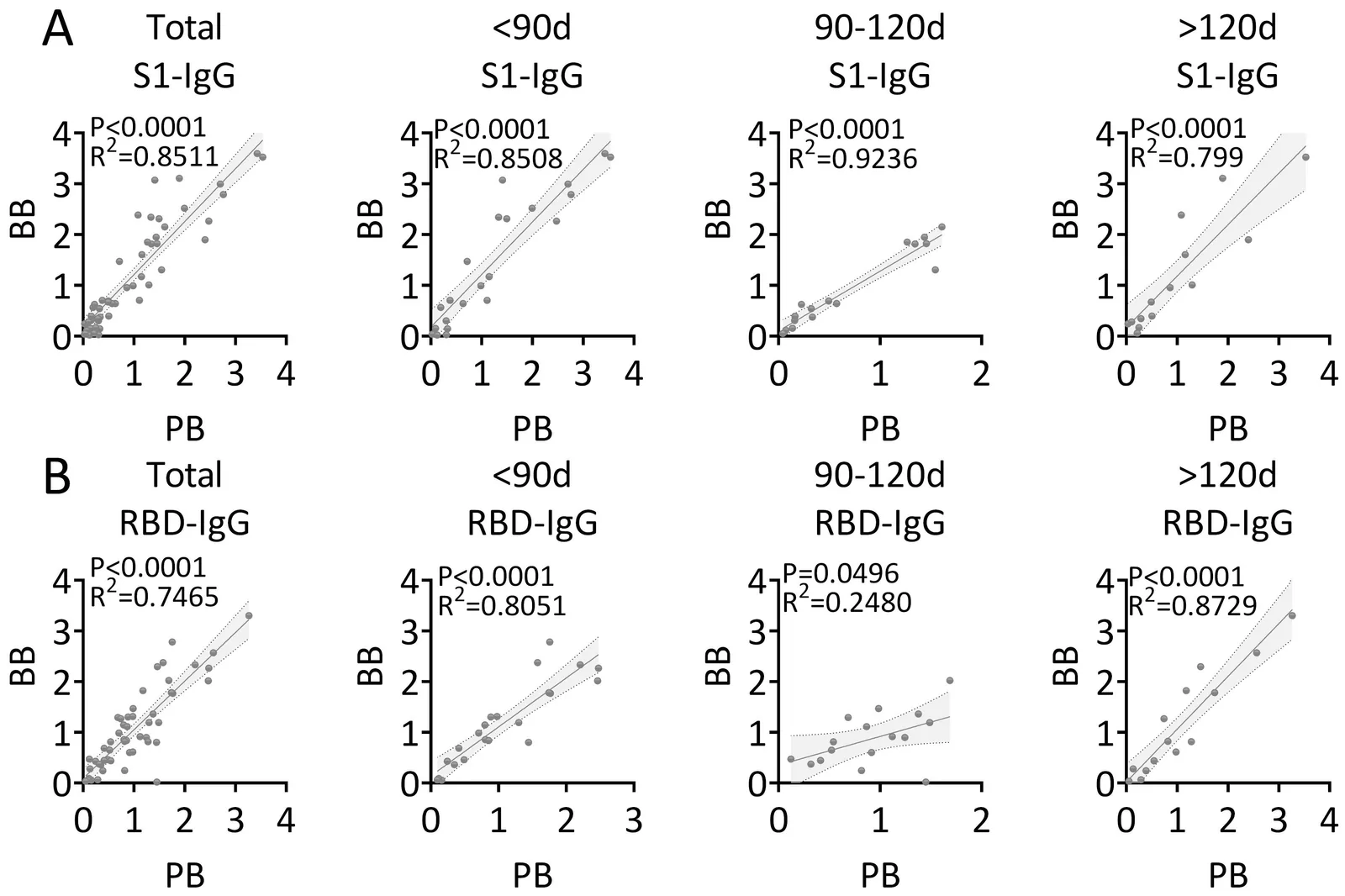
Correlation between maternal and cord blood SARS-CoV-2-specific IgG levels after infection during pregnancy. Correlation analyses were performed between SARS-CoV-2-specific IgG levels in paired maternal peripheral blood and cord blood samples. IgG levels were measured by ELISA and expressed as optical density (OD) values. Pregnant women were grouped according to the infection-to-delivery interval: <90 days, 90–120 days, and >120 days. **(A)** Correlation between maternal peripheral blood and cord blood S1-IgG OD values in the overall cohort and within each infection-to-delivery interval group. **(B)** Correlation between maternal peripheral blood and cord blood RBD-IgG OD values in the overall cohort and within each infection-to-delivery interval group. PB, maternal peripheral blood; BB, cord blood. The solid line represents the fitted regression line, and the shaded area represents the 95% confidence interval. P values and R² values are shown in each panel.

For RBD-IgG (**Figure 6B**), maternal peripheral blood and cord blood levels were also significantly positively correlated in the overall cohort (P<0.0001, R²=0.7465). Subgroup analysis showed strong positive correlations in the <90-day group (P<0.0001, R²=0.8051) and the >120-day group (P<0.0001, R²=0.8729). In the 90-120-day group, the correlation remained statistically significant but was relatively weaker (P = 0.0496, R²=0.2480), suggesting greater variability in the maternal-fetal correspondence of RBD-IgG during this interval.

Taken together, these results indicate that SARS-CoV-2-specific IgG generated in the maternal circulation can be effectively reflected in the fetal circulation via placental transfer. Although the transfer ratios of S1-IgG and RBD-IgG did not show obvious time-dependent differences, the strong positive correlations between maternal and cord blood IgG levels support preserved maternal-fetal IgG transfer after SARS-CoV-2 infection during pregnancy. Compared with RBD-IgG, S1-IgG showed more consistently strong maternal-fetal correlations across all infection-to-delivery intervals, suggesting possible antigen-specific differences in IgG persistence or transfer correspondence.

## Discussion

T lymphocytes play an essential and irreplaceable role in maintaining immune homeostasis and antiviral defense (22). Characterizing their functional status in the context of pregnancy is therefore critical for understanding maternal-fetal immune regulation. Although previous studies have suggested that the immune system during pregnancy undergoes selective regulation (27), systematic studies of the temporal dynamics of post-infection immune responses remain limited. In this study, we performed a multidimensional integrated analysis of maternal cellular and humoral immunity in women with different infection-to-delivery intervals, revealing stage-dependent immune response patterns after SARS-CoV-2 infection during pregnancy. Overall, our results suggest that pregnancy does not lead to global immune suppression; rather, it maintains dynamic balance through structural remodeling and functional regulation.

At the cellular immune level, we observed that antigen-specific T cells retained the ability to secrete IFN-γ at delivery, suggesting that infection-induced cellular immune memory can persist during pregnancy (18). This finding supports the concept of immune adaptation rather than immune suppression during pregnancy (28), indicating that maternal immunity can preserve pathogen-responsive potential while maintaining immune tolerance. Further analysis showed that IFN-γ responses reached a relatively high level 90–120 days after infection, suggesting that this interval may correspond to a functional enhancement stage during the transition from effector T cells to memory T cells (9). We speculate that, under the regulatory influence of pregnancy, this transition is not diminished but may instead follow a more gradual and controlled trajectory. These findings emphasize that the time dimension should be considered when assessing immune status after infection during pregnancy.

The important feature of the present study is that it used antigen-induced IFN-γ production as a functional readout rather than relying solely on immune cell frequencies. This is particularly relevant in pregnancy, where immune cell proportions may be influenced by physiological changes, while functional responsiveness more directly reflects antiviral potential. The observation that the 90-120-day group showed relatively enhanced IFN-γ responses before and after Omicron BA.5.2 antigen stimulation suggests a temporal window in which maternal T cells may retain both basal readiness and antigen-specific reactivity. This provides a functional basis for understanding why simple measurements of infection history or antibody level alone may not fully capture the immune status of pregnant women after SARS-CoV-2 infection.

Second, T-cell subset composition showed marked time-dependent remodeling. Specifically, Th-cell and Tfh-cell subsets were relatively increased during the early post-infection period (<90 days), followed by gradual stabilization at later time points. Tfh cells are important regulators of humoral immune responses, and their early expansion may reflect the immune demand for initiating antibody responses (29). At the same time, the proportion of central memory CD4^+^ T cells increased during the middle and later stages, while functional effector T cells tended to decline, suggesting a transition from effector-dominant responses toward memory maintenance (30). The coexistence of functional and senescent phenotypes among CD8^+^ T cells further indicates that cytotoxic responses and immune regulatory mechanisms may operate in parallel during pregnancy (31). Such coexistence of effector and regulatory states may be an important basis for balancing viral control with avoidance of excessive inflammatory injury.

The humoral immune response showed a pattern different from that of cellular immunity. We observed differences in B-cell and plasma-cell proportions across time groups, suggesting that the humoral compartment is relatively activated during the early phase after infection (32). However, antibody levels, especially IgG levels, did not vary significantly among time groups, indicating that changes in cellular composition do not necessarily translate into synchronous fluctuations in antibody titers. IgM levels generally declined with time, consistent with its classic role as an acute-phase antibody (33). Its low level in cord blood further confirmed that IgM is not efficiently transported across the placenta. In contrast, IgG, the major antibody class that can cross the placenta (34), showed a correlation between maternal and cord blood levels, but no clear time-dependent difference was observed. This suggests that placental transfer efficiency may be regulated more by placental functional status, Fc receptor expression, and the local inflammatory environment than by infection timing alone.

The paired analysis of maternal and cord blood antibodies is another distinctive aspect of this study. Our data showed that cord blood IgM remained low, whereas maternal and cord blood IgG levels were positively correlated. This pattern is consistent with preserved transplacental IgG transfer and does not support overt intrauterine SARS-CoV-2 infection in the studied cohort. Notably, RBD-IgG and S1-IgG did not show identical maternal-fetal distribution patterns, implying that antigen specificity may influence antibody persistence or transfer characteristics. Although this study measured binding antibodies rather than neutralizing activity, the paired maternal-cord design provides useful insight into fetal passive immune acquisition after maternal infection.

The major contribution of this study is that it integrates cellular and humoral immune features from a temporal perspective and proposes a stage-dependent model of immune responses after SARS-CoV-2 infection during pregnancy. Specifically, the immune system may undergo a continuous process from early activation to mid-stage remodeling and late maintenance after infection. In the early phase, Tfh and effector T cells are relatively expanded; in the middle phase, IFN-γ function is enhanced and memory cells are being established; and in the later phase, a relatively stable immune memory state is formed. This model moves beyond static single-time-point analysis and provides a more dynamic framework for understanding immune regulation during pregnancy.

From a clinical perspective, these time-dependent features may have potential relevance. First, incorporating the infection-to-delivery interval into perinatal immune assessment may help explain individual differences in immune indicators more accurately. Second, the relatively enhanced cellular immune function observed at 90–120 days suggests that this interval may be informative for monitoring high-risk pregnant women. Third, the positive correlation between maternal and cord blood IgG levels suggests that optimization of maternal immune status, such as through vaccination strategies, may help improve passive immune protection in neonates. However, because no clear time-dependent difference in antibody transfer was observed, infection timing alone may not be sufficient to predict neonatal immune protection.

These findings also have implications for study design in perinatal infectious disease immunology. Many clinical studies group pregnant women by infection status or disease severity alone. Our results suggest that the interval between infection and delivery should be treated as a biologically meaningful variable, especially when interpreting T-cell function, memory differentiation, and antibody transfer. Future studies combining longitudinal sampling, neutralizing antibody assays, placental Fc receptor analysis, and fetal-neonatal follow-up may further clarify how maternal infection timing influences neonatal immune protection and susceptibility to infection.

This study has several limitations. First, because this was a cross-sectional study, longitudinal changes in the same individuals could not be determined. Second, data analyses were mainly based on the samples of peripheral blood and cord blood. Therefore, the data could not directly reflect the local placental immune microenvironment. Third, no contemporaneous uninfected pregnant control group was available. Accordingly, the present study focused on temporal differences among infected pregnant women according to the interval between infection and delivery, rather than on absolute changes from an uninfected pregnancy baseline. In addition, all participants had received COVID-19 vaccination before infection, which indicates the real-world vaccination background of the study population. Therefore, our findings exhibit the immune responses following natural SARS-CoV-2 infection in previously vaccinated pregnant women. However, because pre-infection samples were unavailable, the respective contributions of vaccination and natural infection could not be clearly distinguished. The sample size also limited trimester-stratified and other subgroup analyses, and some potential clinical and immunological confounding factors could not be fully evaluated. Finally, the immunophenotypic and functional analyses were conducted by using the marker panels and assay controls established in the original study design. Accordingly, the related findings should be interpreted within the scope of the available experimental data and further validated in future studies using more comprehensive approaches. In the future, the longitudinal studies incorporating larger cohorts, uninfected pregnant controls, placental samples, and more comprehensive immune profiling are needed to further validate these findings. Nevertheless, the present study provides a systematic description of maternal and cord-blood immune characteristics at different intervals between infection and delivery and offers a useful basis for subsequent research.

In conclusion, by integrating antigen-specific T-cell function, immune cell subset distribution, antibody levels, and maternal-fetal transfer characteristics, this study systematically depicts the temporal dynamics of immune responses after SARS-CoV-2 infection during pregnancy. The results show that the immune system during pregnancy can establish effective antiviral responses while maintaining tolerance and can provide passive protection to the fetus through IgG transfer. Compared with analyses based on a single time point or a single immune indicator, this study highlights the central role of time in perinatal immune assessment and provides a theoretical basis for optimizing immune monitoring and maternal-neonatal protection strategies.

## Data Availability

The raw data supporting the conclusions of this article will be made available by the authors, without undue reservation.
