# Supplementary material for: Dynamic γδ T-cell Receptor remodeling and antigen-induced T-cell IFN-γ responses after SARS-CoV-2 infection during pregnancy: insights for γδ T-cell immunotherapy
Source: Front Immunol. 2026 Sep 14;17:1914308. doi: 10.3389/fimmu.2026.1914308 (PMC13617096; doi:10.3389/fimmu.2026.1914308)
Supplement: Supplementary file 1 [file DataSheet1.docx]

Supplementary Material

# 1 Supplementary Figures and Tables

## 1.1 Supplementary Figures

**Supplementary Figure 1**

**Gating strategies for CD4⁺ and CD8⁺ T cells, NK cells, γδ T cells, and their phenotypic subsets.** (A) Lymphocytes were selected on an FSC-A-versus-SSC-A plot, singlets on an FSC-A-versus-FSC-H plot, and CD3⁺ T cells were subsequently identified. CD4⁺ and CD8⁺ T cells were defined on a CD4-versus-CD8 plot as CD3⁺CD4⁺CD8⁻ and CD3⁺CD4⁻CD8⁺ cells, respectively, with CD4⁺CD8⁺ double-positive events excluded. The downstream panels show the CD4⁺ and CD8⁺ T-cell subsets and phenotypes analyzed in this study, including the indicated Tfh, Th, Tc, Treg, differentiation, and functional-marker populations. (B) NK cells and their analyzed subsets were identified after lymphocyte and singlet gating. The CD3/CD56, CD94/KIR, and CD56-based gates are shown; CD3⁺CD56⁺ cells are designated NKT-like cells. (C) γδ T cells were identified as TCRγδ⁺ events within CD3⁺ T cells, followed by Vδ1/Vδ2 subdivision and assessment of NKG2D, NKp46, PD-1, and NKp30 expression.

**Supplementary Figure 2**

**Gating strategies for multiparametric flow cytometry analysis of maternal peripheral blood immune cells.** All panels begin with FSC-A/SSC-A lymphocyte-region gating and FSC-A/FSC-H singlet discrimination. (A) CD4⁺ T-cell subsets, including CD4⁺CXCR5⁺PD-1⁺ circulating Tfh-like cells, CD4⁺CD25⁺CD127low/⁻ phenotypic Treg-like cells and CD45RA/CCR7-defined differentiation phenotypes. (B) CD8⁺ T-cell subsets, including CD28 and CD57 gates and CD45RA/CCR7-defined differentiation phenotypes. (C) CD3⁻CD56⁺ NK cells subdivided by relative CD56 intensity and CD3⁺CD56⁺ NKT-like cells; the latter were not interpreted as bona fide invariant NKT cells. (D) CD3⁺TCRγδ⁺ cells and their Vδ1⁺ and Vδ2⁺ subsets, with representative NKG2D, NKP30, NKP46, and PD-1 gates.

**Supplementary Figure 3**

**Gating strategy for IFN-γ-producing T cells after SARS-CoV-2 Omicron BA.5.2 antigen stimulation.** In stimulated PBMC samples, lymphocytes were selected on an FSC-A-versus-SSC-A plot, followed by live-cell and CD3⁺ T-cell gates. CD4⁺ and CD8⁺ T-cell populations were then identified, and IFN-γ⁺ events were quantified within total CD3⁺ T cells and the corresponding CD4⁺ and CD8⁺ T-cell compartments.

## 1.2 Supplementary Tables

**Supplementary Table1**

|  | **<90d (*n*=22)** | **90-120d (*n*=16)** | **＞120d (*n*=14)** | ***χ*^2^/*F*** | ***P*** |
| --- | --- | --- | --- | --- | --- |
| Complications in Pregnancy (%) |  |  |  |  |  |
| Abortion | 0 | 0 | 7.14 | 2.552 | 0.279 |
| GDM | 13.63 | 15.38 | 28.57 | 1.378 | 0.502 |
| Hypertensive disorders | 4.55 | 0 | 0 | 1.253 | 0.545 |
| Preterm birth | 0 | 0 | 0 | - | - |
| Placental abnormalities | 0 | 0 | 0 | - | - |
| Premature rupture of placenta | 9.09 | 15.38 | 14.28 | 0.377 | 0.828 |
| Postpartum Hemorrhage | 9.09 | 0 | 0 | 2.559 | 0.278 |
| Fetal complications (%) |  |  |  |  |  |
| Birth weight | 3050.00(2987.50～3375.00) | 2953.85±390.76 | 3146.43±312.23 | 2.052 | 0.358 |
| FGR/SGA | 9.09 | 38.46 | 7.14 | 6.370 | 0.041 |
| Apgar score 0-5 points | 0 | 0 | 0 | - | - |
| Rate of referral to pediatrics | 13.63 | 30.77 | 5.00 | 2.966 | 0.227 |
| Neonatal hyperbilirubinemia | 11.76 | 11.11 | 0 | 0.956 | 0.620 |

Pregnant women with SARS-CoV-2 infection were grouped according to the interval between infection and delivery: <90 days, 90–120 days, and >120 days. Pregnancy-related complications and fetal/neonatal outcomes were compared among the three groups. Categorical variables are presented as percentages, and continuous variables are presented as mean ± SD or median (interquartile range), as appropriate. The χ²/F value and corresponding P value indicate intergroup comparisons. P < 0.05 was considered statistically significant.

**Abbreviations:** GDM, gestational diabetes mellitus; FGR, fetal growth restriction; SGA, small for gestational age; PROM, premature rupture of membranes; PPROM, preterm premature rupture of membranes.

**Notes:** Placental abnormalities included placenta previa, placental adhesion, and placental abruption. Premature rupture of membranes included PROM and PPROM.

**Supplementary Table 2**

|  | **<90d (*n*=22)** | **90-120d (*n*=16)** | **＞120d (*n*=14)** | ***χ*^2^/*F*** | ***P*** |
| --- | --- | --- | --- | --- | --- |
| WBC (x10^9/L) | 12.04(8.55～15.07) | 12.06±3.95 | 10.68±3.30 | 0.677 | 0.713 |
| RBC (x10^12/L) | 4.15±0.45 | 4.00±0.43 | 3.90±0.36 | 1.557 | 0.222 |
| HBG (g/L) | 122.05±11.67 | 122.15±10.92 | 118.86±11.79 | 0.393 | 0.677 |
| NEUT  LYMPHN  MONO  EO | 0.76(0.73～0.82)  0.14±0.06  0.09(0.06～0.09)  0.01(0～0.01) | 0.78±0.06  0.15±0.05  0.06±0.15  0(0～0.01) | 0.77±0.04  0.16±0.05  0.07±0.02  0±0 | 0.471  0.700  1.667  0.891 | 0.790  0.502  0.200  0.641 |
| BASO | 0(0～0) | 0(0～0) | 0±0 | 1.461 | 0.482 |
| NEUT# (x10^9/L)  LYMPHN# (x10^9/L)  MONO# (x10^9/L) | 9.63(6.33～12.22)  1.46±0.44  0.87±0.36 | 9.65±3.77  1.63±0.33  0.72(0.55～0.87) | 8,25±2.73  (6.33～12.22)  0.79±0.40 | 0.811  1.764  2.046 | 0.666  0.414  0.359 |
| EO# (x10^9/L) | 0.04(0.02～0.07) | 0.04±0.03 | 0.05(0.03～0.07) | 0.605 | 0.739 |
| BASO# (x10^9/L) | 0.02(0.01～0.02) | 0.02±0.01 | 0.02(0.01～0.03) | 0.763 | 0.683 |

Maternal peripheral blood routine hematological parameters were compared among pregnant women grouped according to the interval between SARS-CoV-2 infection and delivery: <90 days, 90–120 days, and >120 days. Data are presented as mean ± SD or median (interquartile range), as appropriate. The χ²/F value and corresponding P value indicate intergroup comparisons. P < 0.05 was considered statistically significant.

**Abbreviations:** WBC, white blood cell count; RBC, red blood cell count; HBG, hemoglobin; NEUT, neutrophils; LYMPHN, lymphocytes; MONO, monocytes; EO, eosinophils; BASO, basophils. The symbol “#” indicates absolute cell count.

**Supplementary Table 3**

|  | **<90d (*n*=22)** | **90-120d (*n*=16)** | **＞120d (*n*=14)** | ***χ*^2^/*F*** | ***P*** |
| --- | --- | --- | --- | --- | --- |
| TCO2 (mmol/L) | 23.64±2.68 | 22.69±2.73 | 23.01±3.20 | 1.055 | 0.375 |
| Lac (mmol/L) | 3.68±1.84 | 4.24±1.21 | 2.80(1.47～3.61) | 4.342 | 0.227 |
| pH | 7.28±0.06 | 7.28±0.09 | 7.29±0.06 | 3.812 | 0.282 |
| PO2 (mmHg)  PCO2 (mmHg)  BE (mmol/L)  SO2 (%) | 25.61±7.22  48.93(41.10～58.45)  -4.68(-7.00～-3.45)  48.70±21.03 | 24.79±9.58  46.89±9.11  -5.54±3.03  47.45±20.43 | 29.57±6.69  45.99±7.26  -5.12±2.94  50.99±17.96 | 4.513  5.596  1.062  2.336 | 0.007  0.133  0.786  0.084 |
| HCO3 (mmol/L) | 22.17±2.46 | 21.28±2.55 | 21.59±2.97 | 0.863 | 0.466 |
| G6PD (U/gHb)  TSH (mlU/L) | 6.96(6.38～7.73)  3.49(1.96～4.28) | 5.78±1.19  3.04±1.55 | 5.66±1.22  3.16±1.94 | 16.115  2.119 | 0.001  0.108 |
| Phe (mg/dl) | 0.71±0.28 | 0.89(0.53～0.87) | 0.91±0.28 | 7.514 | 0.057 |
| 17-OHP (nmol/L) | 5.08±2.44 | 4.71(3.05～6.58) | 5.51(2.95～8.80) | 0.029 | 0.999 |

Umbilical cord blood gas parameters and neonatal metabolic disease screening indicators were compared among neonates born to pregnant women grouped according to the interval between SARS-CoV-2 infection and delivery: <90 days, 90–120 days, and >120 days. Data are presented as mean ± SD or median (interquartile range), as appropriate. The χ²/F value and corresponding P value indicate intergroup comparisons. P < 0.05 was considered statistically significant.

**Abbreviations:** TCO₂, total carbon dioxide; Lac, lactate; PO₂, partial pressure of oxygen; PCO₂, partial pressure of carbon dioxide; BE, base excess; SO₂, oxygen saturation; HCO₃, bicarbonate; G6PD, glucose-6-phosphate dehydrogenase; TSH, thyroid-stimulating hormone; Phe, phenylalanine; 17-OHP, 17-hydroxyprogesterone.
